# Virulence of *Cryptococcus* sp. Biofilms *In Vitro* and *In Vivo* using *Galleria mellonella* as an Alternative Model

**DOI:** 10.3389/fmicb.2016.00290

**Published:** 2016-03-09

**Authors:** Tatiane Benaducci, Janaina de C. O. Sardi, Natalia M. S. Lourencetti, Liliana Scorzoni, Fernanda P. Gullo, Suélen A. Rossi, Jaqueline B. Derissi, Márcia C. de Azevedo Prata, Ana M. Fusco-Almeida, Maria J. S. Mendes-Giannini

**Affiliations:** ^1^Laboratório de Micologia, Departamento de Análises Clínicas, Faculdade de Ciências Farmacêuticas de Araraquara, Universidade Estadual PaulistaAraraquara, Brazil; ^2^Embrapa Brasileira de Agropecuária, Juiz de ForaBrazil

**Keywords:** *Cryptococcus* sp., biofilms, virulence, *Galleria mellonella*

## Abstract

*Cryptococcus neoformans* and *C. gattii* are fungal pathogens that are most commonly found in infections of the central nervous system, which cause life-threatening meningoencephalitis and can grow as a biofilm. Biofilms are structures conferring protection and resistance of microorganism to the antifungal drugs. This study compared the virulence of planktonic and biofilm cells of *C. neoformans* and *C. gattii* in *Galleria mellonella* model, as well as, the quantification of gene transcripts *LAC1*, *URE1*, and *CAP59* by real time PCR. All three of the genes showed significantly increased expressions in the biofilm conditions for two species of *Cryptococcus*, when compared to planktonic cells. *C. neoformans* and *C. gattii* cells in the biofilm forms were more virulent than the planktonic cells in *G. mellonella*. This suggests that the biofilm conditions may contribute to the virulence profile. Our results contribute to a better understanding of the agents of cryptococcosis in the host-yeast aspects of the interaction.

## Introduction

Invasive fungal infections (IFIs) are of global importance. An increasing number of cases have been observed in recent years, and despite current antifungal therapy, mortality rates are high (Perfect, 2013). Among the pathogens causing IFIs, the yeast *Cryptococcus neoformans* is the most prominent in infections of immunocompromised individuals, but *C. gattii* can also cause infections in immunocompetent persons (Tortorano et al., 2012; Perfect, 2013; Prates et al., 2013). The number of cases of cryptococcosis has increased exponentially in the last 30 years due to the advent of AIDS, the use of immunosuppressive therapy in transplant patients and the use of chemotherapeutic agents (McClelland et al., 2013). The use of antiretroviral therapy has been shown to be important in the diagnosis of cryptococcal meningitis in HIV patients, because increasing of CD4 creates benefits for early diagnosis of cryptococcosis through serology (Rajasingham and Boulware, 2015). Although the most serious disease manifestation is meningoencephalitis, cryptococcal pneumony is underdiagnosed and may disseminate to the central nervous system (CNS) and other sites, depending upon host defenses (Brizendine et al., 2011).

*Cryptococcus neoformans* remains the most prevalent human pathogen in this genus and is found in the environment worldwide. Formerly, *C. gattii* was reported mainly in Australia and other subtropical and tropical areas, where it was linked to eucalyptus trees (Smith and Kauffman, 2012). Approximately 1 million cases of cryptococcosis are reported annually, resulting in 600,000 deaths per year (Stie and Fox, 2012; Zhu et al., 2012) and approximately one third of cases (Pyrgos et al., 2013) were related to non-HIV (Bratton et al., 2012; Brizendine et al., 2013). In Brazil, cryptococcosis has recently been identified as the most fatal mycosis in AIDS patients (Albuquerque and Rodrigues, 2012).

The mechanisms of pathogenicity and virulence factors correspond, respectively, to the strategies of the organism or its products to contribute to its virulence. The virulence factors of infectious agents are complex and multifactorial. Polysaccharide synthesis; the capsule; melanin; ability to growth at 37°C; extracellular enzymes such as laccase, phospholipase B, and urease; and biofilm formation are considered to be virulence factors for *Cryptococcus* sp. (Casadevall and Pirofski, 2001; Robertson and Casadevall, 2009; Zaragoza et al., 2009; Kronstad et al., 2011).

Biofilms are communities of microorganisms involving an extracellular matrix attached to a solid surface, whose development provides important benefits such as increased nutrient concentrations in the biofilm-liquid interface. The polymer matrix promotes the adsorption of nutrient molecules and provides protection from environmental insults (pH changes, salt concentration, dehydration, aggressive chemicals, bactericides, antibiotics, predators, lytic bacteria, and heavy metals). Thus, biofilm microorganisms differ profoundly from planktonic cells but retain their invasiveness and ability to evade the host immune system (Costerton et al., 1995; Sardi et al., 2013). *C. neoformans* is able to form biofilm on medical devices, including ventriculoatrial shunt catheters (Walsh et al., 1986; Bach et al., 1997), peritoneal dialysis fistula (Braun et al., 1994) and prosthetic cardiac valves (Banerjee et al., 1997), that highlight the ability of this organism to adhere to medical devices. Normally, patients who do not benefit from antifungal therapy and patients who present serious visual loss or ocular palsies or remain with high cerebrospinal fluid pressure levels, must be considered for ventriculoperitoneal placement (Corti et al., 2014). The increasing use of shunts to manage intracranial hypertension associated with cryptococcal meningoencephalitis suggests the importance of investigating the biofilm-forming properties of this organism (Bach et al., 1997) apud (Martinez and Casadevall, 2015).

Non-conventional animal models of infection can be used to investigate the virulent traits of a pathogen or the therapeutic efficacy of a drug, as well as the host–pathogen interactions. The *Galleria mellonella* model represents a versatile experimental system to study fungal virulence and antifungal efficacy (Mylonakis et al., 2005; Fuchs and Mylonakis, 2006; Scorzoni et al., 2013).

The objective of this study was to evaluate the ability of *C. neoformans* and *C. gattii* in biofilm and planktonic forms in *G. mellonella*, an alternative model, and to relatively quantify the gene transcripts involved in virulence by real time PCR.

## Materials and Methods

### Microorganisms

*Cryptococcus neoformans* var. *grubii* ATCC 90112, serotype A, molecular type VNI and *C. gattii* ATCC 56990, serotype BC, molecular type VGIII, belonging to the mycology collection of the Laboratory of Clinical Mycology, Department of Clinical Analysis, Faculty of Pharmaceutical Sciences, UNESP, Araraquara were used in this study. Yeasts were kept frozen in glycerol and subcultured at the time of the experiment.

### Biofilm Formation

Both *C. neoformans* and *C. gattii* were grown in Sabouraud dextrose broth (Difco Laboratories, Detroit, MI, USA) with shaking (200 rpm) at 37°C until the late log phase (as determined by a growth curve constructed from absorbance readings at an optical density of 600 nm). Biofilm formation was performed according to Martinez and Casadevall (2005), with some modifications. The cells were collected by centrifugation, washed twice with phosphate-buffered saline (PBS), counted with a hemocytometer, and suspended at 1 × 10^8^ cells/mL in PBS. For each microorganism, 100 μL of the suspension was added into individual polystyrene wells in 96-well plates (TPP Trasadingen, Switzerland), and the plates were incubated at 37°C without shaking for 2 h for the adhesion stage. The mature biofilms were formed for 72 h with shaking at 70 rpm at 37°C in RPMI-1640 (Sigma–Aldrich, St. Louis, MO, USA) that contained L-glutamine, but not sodium bicarbonate, was supplemented with 2% glucose, and was buffered to pH 7.0 using 0.165 M MOPS, (Sigma–Aldrich, St. Louis, MO, USA). The wells without *Cryptococcus* cells were used as controls. Following the adhesion stage, the wells containing *Cryptococcus* biofilms were washed three times with 0.05% Tween 20 in PBS to remove non-adhered cryptococci. Fungal cells that remained attached to the plastic surface were considered true biofilms. All assays were carried out in triplicate.

### Scanning Electron Microscopy (SEM)

*Cryptococcus* sp. biofilms were grown on glass coverslips in microtiter plates with RPMI-1621 for 72 h. Coverslips with biofilms were then washed three times with PBS and transferred to another microtiter plate containing 2.5% glutaraldehyde and incubated for 48 h at 4°C. The samples were serially dehydrated in alcohol, fixed in a critical-point drier (Samdri-790; Tousimis, Rockville, MD, USA), coated with gold-palladium (Desk-1; Denton Vacuum, Inc., Cherry Hill, NJ, USA), and viewed with a JEOL (Tokyo, Japan) JSM-6400 scanning electron microscope at a voltage of 10.0 kV; the image was captured at 10,000×.

### Confocal Laser Scanning Microscopy (CLSM)

Confocal microscopy was performed according to Martinez and Casadevall, 2005. Mature biofilms were incubated for 45 min at 37°C in a solution of CAAF 488 (Concanavalin A conjugated to Alexa Fluor 488, CAAF; Molecular Probes, USA) and FUN 1 (Molecular Probes, USA). Thus, 4 μL of FUN 1 (10 mM) and 15 μL of CAAF 488 (5 mg/mL) were added to 3 mL of sterile PBS to obtain 10 μg/mL FUN 1 and 25 μg/mL CAAF. Subsequently, the coverslips were washed with distilled water taken from the wells and inverted over 4 μL of Fluoromount-G (Sigma–Aldrich, USA) that was previously deposited on microscope slides for observation under confocal microscopy (LSM 510 META, Zeiss). These trials were conducted in collaboration with the Oswaldo Cruz Institute in Rio de Janeiro, Department of Cell Biology, under the coordination of Professor Dr. Marcelo Machado Pelajo.

### Metabolic Activity of the Biofilm

The metabolic activity of the biofilms was evaluated according to (Martinez and Casadevall, 2006b). Semi-quantitative measurements of planktonic cells, biofilm formations and mature biofilms of *C. neoformans* and *C. gattii* were obtained from the reduction assay of 2,3-bis (2-methoxy-4-nitro-5-sulfophenyl)-5-[carbonyl (phenylamino)]-2H-tetrazolium hydroxide (XTT). The metabolic activity of the cells was measured by mitochondrial dehydrogenase activity, which reduces the XTT tetrazolium salt to formazan salt, resulting in a colorimetric change. For this assay, 50 μL of XTT solution (1 mg/mL in PBS) and 4 μL of a solution of menadione (1 mM in acetone, Sigma Chemical Co.) were added to each well. The microplates were incubated at 37°C for 5 h and measured at 490 nm (Microplate Reader iMark TM, BIO-RAD). In all experiments, RPMI was included as a negative control.

### Antifungal Activity of Amphotericin B

The antifungal activity of amphotericin B (AMB) against planktonic cells and biofilm of *C. neoformans* and *C. gattii* was evaluated by metabolic activity quantification. AMB was tested in range of concentration equal to 0.0625–128 μg/mL for 48 h of treatment and the fungicidal concentration (FC) was considered the lower concentration to metabolic activity below 10% (Martinez and Casadevall, 2006b). This assay was performed XTT assay as previously described.

### RNA Isolation and cDNA Synthesis

Total RNA was extracted from *Cryptococcus* sp. in planktonic and biofilm formations using TRIZOL^®^reagent (Invitrogen, Carlsbad, CA, EUA, USA) according to the manufacturer’s instructions. The total RNA from the two situations was treated with DNase I (Invitrogen, Carlsbad, CA, USA); its concentration and purity were then determined using a spectrophotometer (GE, Nanovue Plus). The integrity of the RNA was verified by electrophoresis with 1% agarose gels. RNA was converted to cDNA using 1 μg of total RNA and reverse transcriptase (RevertAidTM H Minus Reverse Transcriptase, Fermentas Life Sciences, Canada).

### Real Time PCR of the Genes *LAC1*, *URE1*, and *CAP59* in Planktonic and Biofilm Formations

For the analysis of differential gene expressions by RT-PCR (Real Time PCR), specific primers were used for genes (Supplementary Table S1) encoding proteins that are differentially expressed in *C. neoformans* (ATCC 90112) and *C. gattii* (ATCC 56990) strains in planktonic and biofilm formations. Primers were used at 0.8 μM each for specific forward and reverse primer and cDNA at 20 ng in 20 μl reactions. The test was performed by Maxima SYBR Green/ROX qPCR Master Mix (2X) (Thermo Scientific), and the *GAPDH* (Glyceraldehyde 3-phosphate dehydrogenase) gene was used as a reference gene. The reaction program was 50°C for 2 min, 95°C for 10 min, and 40 cycles of 95°C for 15 s, and annealing and synthesis occurred at 60°C for 1 min. Following PCR, a melting-curve analysis was performed, which confirmed that the signal corresponded to a single PCR product in an Applied Biosystems 7500 cycler. The data were analyzed by the 2^-ΔCT^ method (ΔCt = ΔCt target–ΔCt endogen; Livak and Schmittgen, 2001).

### Virulence of Planktonic and Biofilm Cells in *Galleria mellonella*

*Galleria mellonella* caterpillars were produced by EMBRAPA – Juiz de Fora/MG. Larvae weighing 100–200 mg were selected for the experiment. The day before the experiment, the larvae were stored at 37°C. The biofilms were destroyed with a scraper, and the cells were counted in a hemocytometer chamber together with the planktonic cells for the final concentration of 5 × 10^6^ cells/larvae (cell concentrations previously standardized with planktonic cells). Larvae were infected with 10 μL of inocula injected into the hemocell through the last proleg using a Hamilton syringe (Hamilton, EUA). The groups of *G. mellonella* were incubated at 37°C for 7 days, and their death was monitored daily. The death of the larvae was assessed by the lack of movement after touching them with tweezers. For each condition, a total of 16 larvae were used, and each experiment was repeated at least twice.

### Statistical Analysis

All analyses of the dates were performed with GraphPad Prism 5.0 (GraphPad Software, La Jolla, CA, USA). Survival curves were analyzed by the Log-rank (Mantel-Cox) Test. Real time PCR was performed statistically by *t*-test and *p*-values < 0.05 were considered significant.

## Results

### Biofilm Formation

**FIGURE 1:**
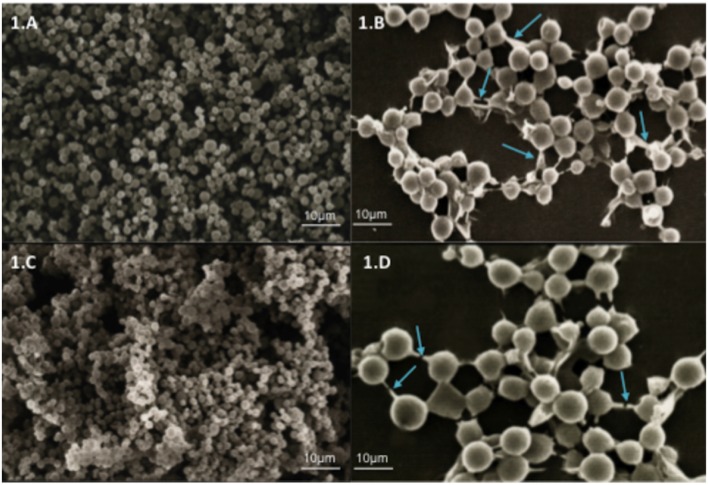
**Scanning electron microscopy (SEM) showing yeast communities adhered to surfaces of glass coverslips indicating the formation of mature biofilms. (A)** Biofilm of *Cryptococcus neoformans* ATCC 90112 at 750× magnification. **(B)** Biofilm of *C. neoformans* ATCC 90112 at 3500× magnification. **(C)** Biofilm of *C. gattii* 56990 at 750× magnification. **(D)** Biofilm of *C. gattii* 56990 at 2500× magnification. The blue arrows indicate the self-produced matrix.

The ability of the *C. neoformans* and *C. gattii* strains to form biofilms *in vitro* was evaluated. Biofilm formation by *Cryptococcus* sp. was demonstrated by SEM. It was observed that every strain was able to form agglomerates of cells characterized by a microorganism community firmly adhered to a non-biological surface and resulting in the formation of a mature biofilm. The SEM data provided useful information on the cell morphology present in the biofilm structure. The biofilms of *C. neoformans* (**Figures 1A,B**) and *C. gattii* (**Figures 1C,D**) strains consist of a dense network of yeasts.

### Confocal Laser Microscopy

**FIGURE 2:**
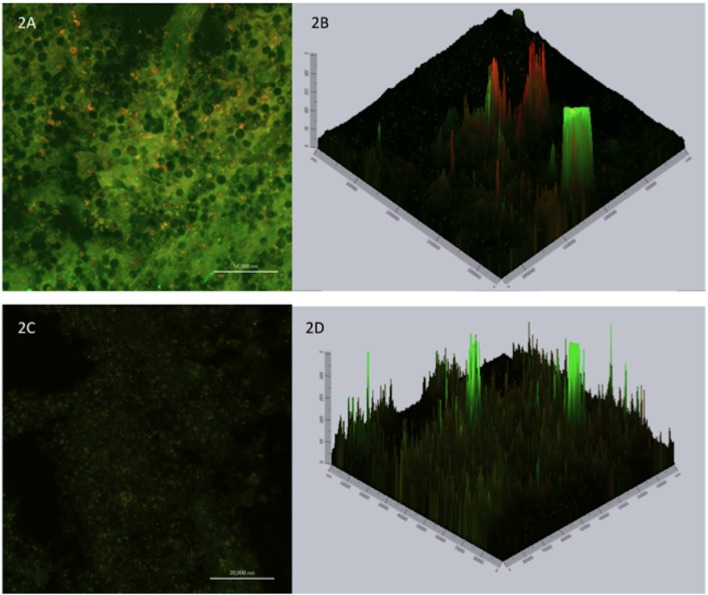
**Confocal laser microscopy showing metabolically active cells in mature biofilms.** Staining with FUN1 fluorochrome shows the cytoplasm of metabolically active cells dyed orange–yellow. Staining with concavalin associated with Alexa Fluor 488 (CAAF) shows the presence of mannose in the cell wall of *Cryptococcus* sp. Orthogonal images show the thickness and architecture of the biofilms. **(A)** Mature biofilm of *C. neoformans* ATCC 90112. **(B)** Thickness of mature biofilm of *C. neoformans* ATCC 90112. **(C)** Mature biofilm of *C. gattii* ATCC 56990. **(D)** Thickness of mature biofilm of *C. gattii* ATCC 56990.

Confocal laser scanning microscopy is useful for determining the viability/activity of the cells adhered to the glass surface and for analyzing the biofilm thickness. Applying two fluorochromes, Concanavalin A conjugated to Alexa Fluor 488 (CAAF) and FUN1, to determine the activity/viability allows the observation of metabolically active structures in fungal cells. In combination, the CAAF specifically binds to cell wall polysaccharides. Images of mature biofilms of *C. neoformans* ATCC 90112 and *C. gattii* ATCC 56990 were acquired by confocal microscopy (**Figures 2A,C**). The yellow-orange staining due to FUN1 is dense in the cytoplasm of metabolically active cell aggregates, while the green coloration results from the binding of Concanavalin A to the glucose and mannose moieties of the fungal cell wall. Orthogonal images of biofilms of the strains were analyzed to determine their thickness and architecture. Sections of the three-dimensional images showed that the biofilm of *C. neoformans* strain ATCC 90112 has a thickness of 10 μm (**Figure 2B**), while *C. gattii* ATCC 56990 (**Figure 2D**) has a thickness of 5.4 μm.

### Kinetics of Biofilm Formation

The kinetics of biofilm formation on polystyrene microdilution plates by both *Cryptococcus* species was compared using the colorimetric XTT reduction assay to determine metabolic activity. During a period of 2 h, the *Cryptococcus* yeast became firmly adhered to the plastic surface. In the intermediate stage (12 h) the fungal population increased to form a cell monolayer, thus initiating the process of biofilm formation. During the maturation stage (24–72 h), fungal growth involves the formation of microcolonies consisting of clustered cells and resulting in compact structures adhering to the plastic surface.

**FIGURE 3:**
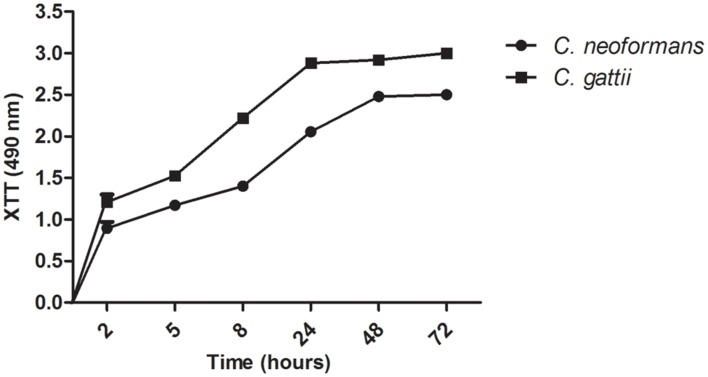
**Kinetics of biofilm formation by *Cryptococcus* sp. in microdilution plates as determined by the XTT reduction assay**.

All samples produced biofilms from 24 to 72 h, and the initial biofilm formation was observed by 2 h of incubation, which includes the period of initial adhesion. Over a period of 12 to 24 h, a continuously increasing biofilm was observed. The kinetics of biofilm formation was similar in both strains analyzed and showed no significant differences (**Figure 3**).

### Antifungal Activity of Amphotericin B

The Supplementary Table S2 shows the values of fungicidal concentration (FC) of AMB for *C. neoformans* and *C. gattii* in planktonic and biofilm conditions. The FC was 0.5 and 0.25 μg/mL for planktonic cells of *C. neoformans* and *C. gattii*, respectively. In the biofilm condition, we observed an increase of the fungicidal concentration for *C. neoformans* as well as *C. gattii*, both with concentration of 64.0 μg/mL.

### Gene Expressions of *LAC1*, *URE1*, and *CAP59* in Biofilms and Planktonic Cells of *Cryptococcus* sp.

**FIGURE 4:**
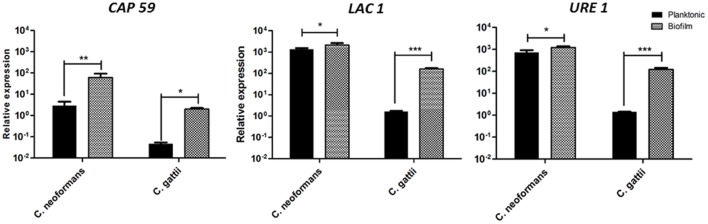
**Expression of the CAP59, laccase (LAC1), and urease (URE1) genes normalized by GAPDH in biofilms and planktonic cells of *C. neoformans* and *C. gattii***. Graphs are plotted in power of 10 to better visualization the results. *p < 0.5, **p < 0.01, and ***p < 0.001.

We used real time PCR assay, with *GAPDH* as the internal control. The results of the relative gene expressions of *URE1*, *LAC1*, and *CAP59* with biofilm and planktonic cells of *C. neoformans* and *C. gattii* are shown in **Figure 4**. All three of the genes showed significantly increased expressions in the biofilm conditions for two species of *Cryptococcus*, when compared to planktonic cells. However, we can see that the increased expression of genes *LAC1* and *URE1* was more relevant when comparing biofilm and planktonic cells of *C. gattii*. On the other hand, the increase in *CAP59* expression in biofilms of *C. neoformans*, when compared with planktonic cells, showed higher significance than other genes.

### Virulence of Planktonic and Biofilm Cells in *Galleria mellonella*

**FIGURE 5:**
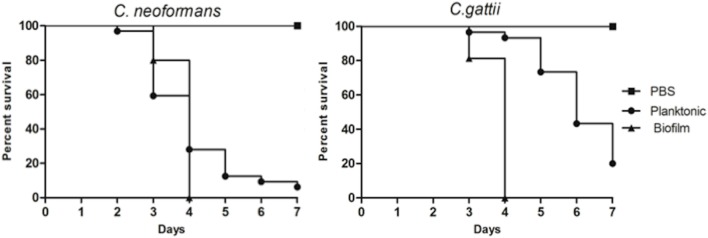
**Comparison of virulence from planktonic and biofilm cells of *C. neoformans* ATCC 90112 and *C. gattii* ATCC 56990 using *Galleria mellonella* as the infection model.** The differences were statistically significant (*p* < 0.05). Graphs represent the average of three repetitions.

To determine an adequate concentration for the virulence tests with planktonic and biofilm cells, survival curves were created for different concentrations of yeast (planktonic). The concentration of 5 × 10^6^ cells/larvae was selected for the experiments (data not shown). The virulence of the biofilm and planktonic cells were then compared *in vivo* in the *G. mellonella* model. After 4 days, all larvae infected with cells from *C. neoformans* and *C. gattii* biofilms were dead. Larvae infected with planktonic cells exhibited 30% live larvae when infected with *C. neoformans* and 80% live larvae when infected with *C. gattii* in the time of 4 days. It is clear that cells from biofilms are more virulent, and in the final part of the experiment (7 days), live larvae still remained. When larvae were infected with planktonic cells, the death rate did not reach 100% in any of the strains tested (**Figure 5**).

## Discussion

Characterizations of the virulence factors of infectious agents have revealed great complexity. These factors can be divided into two categories: those that promote colonization and invasion as biofilms and those that damage the host by the production of hydrolytic enzymes (Mendes-Giannini et al., 2000). Biofilm formation is a common mechanism used by microorganisms to survive in hostile environments and to colonize and establish themselves in new environments, thus conferring protection against their destruction (Hall-Stoodley et al., 2004). Moreover, fungal biofilms are an increasing clinical problem associated with high mortality rates (Williams and Costerton, 2012). The production of melanin (Williamson, 1994), the presence of capsules (Kwon-Chung et al., 1992), growth at 37°C (Kraus et al., 2003), alpha mating type (Kwon-Chung et al., 1982), and the production of phospholipase (Wright et al., 2004), superoxide dismutase (Cox et al., 2003), protein kinases (Alspaugh et al., 1998), and urease (Cox et al., 2000), as well as the synthesis of polysaccharides (Albuquerque et al., 2014), were phenotypically and molecularly characterized as *in vivo* virulence factors related to invasion and survival in the host (Perfect, 2005).

In this study, strains of *C. neoformans* and *C. gattii* were able to form biofilms. These data were confirmed, and electron microscopy showed the formation of a dense network of yeasts with a self-produced extracellular matrix linking the cells of *Cryptococcus* sp. Factors such as the characteristics of the medium and the attachment surface are important for biofilm development by *Cryptococcus* sp., and strains of *C. neoformans* formed denser biofilms when they were cultured in RPMI 1640 medium (Martinez and Casadevall, 2006a). Biofilm formation is the most common growth form of microorganisms in nature; it represents up to 65% of all clinical infections, and given the high level of antimicrobial resistance, biofilms represent a huge problem in clinical practice (Donlan and Costerton, 2002; Ramage and Williams, 2013; Martinez and Casadevall, 2015). Biofilm production by *Cryptococcus* species has often been described as a strategy associated with chronic infection resulting from acquired resistance to host immune mechanisms and antifungal therapy (Martinez et al., 2006).

Amphotericin B activities increased 100X the fungicidal concentration for both species in biofilm condition. Our data corroborate to Martinez and Casadevall (2006a), since the authors showed a lower reduction in metabolic activity of *C. neoformans* biofilms when treated with the AMB in concentrations of 4 and 8 μg/mL, while for planktonic cells, the metabolic activity is reduced significantly after treatment with 0.5 μg/mL of AMB.

Additionally, our results demonstrated increased expression of the *CAP59* gene in *C. neoformans* and *C. gattii* when compared the biofilm situations to the planktonic mode, suggesting that this gene may be related to the virulence of this infection. Although no studies of the *CAP59* gene have been described in biofilms, the importance of this increased expression could be related to virulence and resistance because the presence of the capsule prevents the actions of both phagocytes and antifungal drugs. Few studies have been made of *C. gattii*; the genes that regulate virulence were therefore inferred from the work on *C. neoformans*. Several virulence factors were identified using *C. neoformans* as a model (Perfect, 2005). Some evidence of differences in the regulation of virulence genes between the two species have been published (Hicks and Heitman, 2007; Ngamskulrungroj et al., 2009). Our results demonstrated increased expression of *CAP* genes in the biofilms of *C. neoformans* and *C. gattii.* It has been reported that *C. neoformans* requires four genes to form the capsule: *CAP59*, *CAP64*, *CAP60*, and *CAP10* (Chang et al., 1996; Grijpstra et al., 2009). However, unencapsulated homologs of *CAP10*, *CAP59*, and *CAP64* have been found in other fungi, suggesting that these genes may be involved in other processes (García-Rivera et al., 2004). *CAP59* was the first gene that was directly related to the phenotype of the capsule and to virulence (Chang and Kwon-Chung, 1994), and it is present in all varieties of *C. neoformans* (Alspaugh et al., 1998). Del Poeta (2004) suggested that changes in the capsule structure could be related to responses to specific environmental conditions, with important implications for the host immune response (Del Poeta, 2004).

Our results also demonstrated an increased expression of laccase, due to expression of *LAC1* gene, in the biofilms of *C. neoformans* and *C. gattii* compared to planktonic cells. Laccase, a phenoloxidase, was described by Zhu et al. (2001) in as an important virulence factor contributing to the protection of the fungus against oxidative damage by host phagocytes; it is located in the cell wall of *C. neoformans* (Zhu et al., 2001). The enzyme has also been described for *C. gattii* (Sorrell, 2001) and may also modulate the immune response and contribute to the spread of the disease to the CNS (Qiu et al., 2012).

Urease, codified by the gene *URE1*, is a metalloenzyme that catalyzes the hydrolysis of urea to ammonia and carbamate in physiological conditions and increases the pH. The importance of this enzyme was described in *Helicobacter pylori*, *Proteus mirabilis*, and its clinical significance was described in *C. neoformans, C. gattii, Coccidioides immitis, Histoplasma capsulatum*, *Sporothrix schenckii*, *Trichosporon*, and *Aspergillus* species (Cox et al., 2000). The urease gene was expressed more in the biofilm condition in *C. neoformans* and *C. gattii* than in planktonic cells. Urease may promote the transmigration of the fungus across the blood–brain barrier and facilitate invasion of the CNS, indicating that urease activity can affect the interaction of the fungus with the endothelial cells and brain microcapillaries in the blood–brain barrier (Morrow and Fraser, 2013). Cox et al. (2000) suggested that urease activity in murine models increases the survival of mice infected with the mutant urease-negative gene (Cox et al., 2000), and Feder et al. (2015) demonstrated the importance of this enzyme in the virulence of *C. gattii*. In this work we emphasize, for the first time, the importance of these three genes in the biofilm condition (Feder et al., 2015).

Additionally, the importance of using alternative animal models in the study of virulence is increasing due to advantages such as the absence of ethical issues, cost, and the possibility of using a large number of individuals (Fuchs and Mylonakis, 2006; Desalermos et al., 2012). The use of *G. mellonella* in the study of *Cryptococcus* virulence is well documented (Firacative et al., 2014; Desalermos et al., 2015; Trevijano-Contador et al., 2015). Mylonakis et al. (2002) evaluated the virulence, host immune responses and efficacy of antifungal compounds and considered the *G. mellonella* model suitable for *in vivo* studies of C. *neoformans* (Mylonakis et al., 2002). Fuchs et al. (2010) also described *G. mellonella* as an appropriate model for the study of this yeast (Fuchs et al., 2010) and Desalermos et al. (2015) studied *C. neoformans* and various genes involved in the yeast-host interaction in *C. elegans* and *G. mellonella* (Desalermos et al., 2015). Although insects and mammalians are evolutionary distance, *G. mellonella* presents characteristics that support its use as *in vivo* model for fungal assays. The presence of at least six cellular type in hemolymph including phagocytic cells and the possibility of maintain *G. mellonella* at 37°C to simulate the mammalian temperature are important when considering the use of this alternative animal model in the study of human fungal disease (Kavanagh and Reeves, 2004; Fuchs and Mylonakis, 2006; Desalermos et al., 2012). Moreover, correlations have been found in mammals with respect to virulence and antifungal treatment (Mylonakis et al., 2005; London et al., 2006). Biofilm-producer and non-producer strains of *Candida albicans* were evaluated in *G. mellonella* and showed the biofilm-producer’s high ability to kill larvae (Cirasola et al., 2013).

As part of the characterization of the biofilms of *Cryptococcus* sp., we evaluated the virulence these species in planktonic and biofilm conditions in a *G. mellonella* model. The survival curve revealed that cells from biofilms are more virulent than planktonic cells, and this profile was also observed for *C. neoformans* and *C. gattii*. Microorganisms are protected by the extracellular matrix in biofilms, which is mainly composed of glycoproteins and polysaccharides. In addition to conferring protection and resistance, our results showed that biofilm cells are more virulent and quickly cause death in *G. mellonella* larvae.

Wand et al. (2012) reported that *Acinetobacter baumannii* biofilms could produce higher rates of mortality in the *G. mellonella* model compared to planktonic cells of various clinical isolates. Cirasola et al. (2013) also observed the virulence of clinical isolates of *C. albicans* with or without biofilm capability; the survival of *G. mellonella* larvae following infection was reduced and 80% of the infected larvae died within 72 h.

Our studies revealed that *Cryptococcus* sp. yeast cells that underwent biofilm formation for the first-time were more virulent than cells that have not gone through the same process when analyzed in the *G. mellonella* model. Other virulence factors could be involved during biofilm formation that might help us to understand pathogenic infections in *G. mellonella*. A study conducted by Uppuluri and Lopez-Ribot (2010) showed that cells that detach from a biofilm have a greater association with mortality than those from planktonic microorganisms. The dispersed cells of biofilms are more virulent and cause higher mortality than planktonic cells, which can be explained by epigenetic alterations within the cells that undergo biofilm development (Uppuluri and Lopez-Ribot, 2010; Sardi et al., 2014). Ramage et al. (2012) demonstrated an increased expression of aspartyl proteinases in *C. albicans* biofilms compared with planktonic cells. Their study reported that the production of proteinase by *C. albicans* established adhesion, invasion, and tissue destruction. Their results demonstrate that the expression of proteinases may be related to the severity of disease because the expression of these enzymes was significantly higher in mature biofilms (Ramage et al., 2012). *C. neoformans* also produces various proteases and aspartyl proteinases which have been proposed as potential virulence factors that contribute to host tissue invasion and colonization by the pathogen. The increase in proteases could explain the premature death of the larvae infected with *C. neoformans* and *C. gattii* biofilms.

These preliminary results demonstrate the higher virulence of *C. neoformans* and *C. gattii* biofilms. Additional studies are *needed* to understand the virulence mechanisms of the biofilms involved in the premature death of the larvae.

These preliminary results demonstrate the higher resistance of AMB and virulence in *G. mellonella*, as well as increased expression of virulence genes of *C. neoformans* and *C. gattii* biofilms. Additional studies are needed to understand the virulence mechanisms of the biofilms involved in the premature death of the larvae.

## Author Contributions

All authors listed, have made substantial, direct and intellectual contribution to the work, and approved it for publication.

## Conflict of Interest Statement

The authors declare that the research was conducted in the absence of any commercial or financial relationships that could be construed as a potential conflict of interest.

## Supplementary Material

TABLE S1 **Primers used for RT-pPCR analysis for the virulence analysis**.

TABLE S2 **Fungicidal concentration of amphotericin B for planktonic cells and biofilm madure of *Cryptococcus neoformans* and *Cryptococcus gattii***.

## References

[B1] Albuquerque P. C. Fonseca F. L. Dutra F. F. Bozza M. T. Frases S. Casadevall A. (2014). *Cryptococcus neoformans* glucuronoxylomannan fractions of different molecular masses are functionally distinct. *Future Microbiol.* 9 147–161. 10.2217/fmb.13.163 24571070 PMC4294706

[B2] Albuquerque P. C. Rodrigues M. L. (2012). Research trends on pathogenic *Cryptococcus* species in the last 20 years: a global analysis with focus on Brazil. *Future Microbiol.* 7 319–329. 10.2217/fmb.11.162 22393887

[B3] Alspaugh J. A. Perfect J. R. Heitman J. (1998). Signal transduction pathways regulating differentiation and pathogenicity of *Cryptococcus neoformans*. *Fungal Genet. Biol.* 25 1–14. 10.1006/fgbi.1998.1079 9806801

[B4] Bach M. C. Tally P. W. Godofsky E. W. (1997). Use of cerebrospinal fluid shunts in patients having acquired immunodeficiency syndrome with cryptococcal meningitis and uncontrollable intracranial hypertension. *Neurosurgery* 41 1280–1282. 10.1097/00006123-199712000-00008 9402579

[B5] Banerjee U. Gupta K. Venugopal P. (1997). A case of prosthetic valve endocarditis caused by *Cryptococcus neoformans* var. neoformans. *J. Med. Vet. Mycol.* 35 139–141. 10.1080/02681219780001031 9147274

[B6] Bratton E. W. El Husseini N. Chastain C. A. Lee M. S. Poole C. Stürmer T. (2012). Comparison and temporal trends of three groups with cryptococcosis: HIV-infected, solid organ transplant, and HIV-negative/non-transplant. *PLoS ONE* 8:e43582 10.1371/journal.pone.0043582 PMC3427358 22937064

[B7] Braun D. K. Janssen D. A. Marcus J. R. Kauffman C. A. (1994). Cryptococcal infection of a prosthetic dialysis fistula. *Am. J. Kidney Dis.* 24 864–867. 10.1016/S0272-6386(12)80683-4 7977331

[B8] Brizendine K. D. Baddley J. W. Pappas P. G. (2011). Pulmonary cryptococcosis. *Semin. Respir. Crit. Care Med.* 32 727–734. 10.1055/s-0031-1295720 22167400

[B9] Brizendine K. D. Baddley J. W. Pappas P. G. (2013). Predictors of mortality and differences in clinical features among patients with Cryptococcosis according to immune status. *PLoS ONE* 8:e60431 10.1371/journal.pone.0060431 PMC3608592 23555970

[B10] Casadevall A. Pirofski L. (2001). Host-pathogen interactions: the attributes of virulence. *J. Infect. Dis.* 184 337–344. 10.1086/322044 11443560

[B11] Chang Y. C. Kwon-Chung K. J. (1994). Complementation of a capsule-deficient mutation of *Cryptococcus neoformans* restores its virulence. *Mol. Cell. Biol.* 14 4912–4919. 10.1128/MCB.14.7.4912 8007987 PMC358863

[B12] Chang Y. C. Penoyer L. A. Kwon-Chung K. J. (1996). The second capsule gene of *Cryptococcus neoformans*, CAP64, is essential for virulence. *Infect. Immun.* 64 1977–1983. 8675296 10.1128/iai.64.6.1977-1983.1996 PMC174025

[B13] Cirasola D. Sciota R. Vizzini L. Ricucci V. Morace G. Borghi E. (2013). Experimental biofilm-related *Candida* infections. *Future Microbiol.* 8 799–805. 10.2217/fmb.13.36 23701334

[B14] Corti M. Priarone M. Negroni R. Gilardi L. Castrelo J. Arechayala A. I. (2014). Ventriculoperitoneal shunts for treating increased intracranial pressure in cryptococcal meningitis with or without ventriculomegaly. *Rev. Soc. Bras. Med. Trop.* 47 524–527. 10.1590/0037-8682-0176-2013 25229298

[B15] Costerton J. W. Lewandowski Z. Caldwell D. E. Korber D. R. Lappin-Scott H. M. (1995). Microbial biofilms. *Annu. Rev. Microbiol.* 49 711–745. 10.1146/annurev.mi.49.100195.003431 8561477

[B16] Cox G. M. Harrison T. S. McDade H. C. Taborda C. P. Heinrich G. Casadevall A. (2000). Urease as a virulence factor in experimental cryptococcosis. *Infect. Immun.* 68 443–448. 10.1128/IAI.68.2.443-448.2000 10639402 PMC97161

[B17] Cox G. M. Harrison T. S. McDade H. C. Taborda C. P. Heinrich G. Casadevall A. (2003). Superoxide dismutase influences the virulence of *Cryptococcus neoformans* by affecting growth within macrophages. *Infect. Immun.* 71 173–180. 10.1128/IAI.71.1.173-180.2003 12496163 PMC143417

[B18] Del Poeta M. (2004). Role of phagocytosis in the virulence of *Cryptococcus neoformans*. *Eukaryot. Cell* 3 1067–1075. 10.1128/EC.3.5.1067-1075.2004 15470235 PMC522596

[B19] Desalermos A. Fuchs B. B. Mylonakis E. (2012). Selecting an invertebrate model host for the study of fungal pathogenesis. *PLoS Pathog.* 8:e1002451 10.1371/journal.ppat.1002451 PMC3271057 22319439

[B20] Desalermos A. Tan X. Rajamuthiah R. Arvanitis M. Wang Y. Li D. (2015). A multi-host approach for the systematic analysis of virulence factors in *Cryptococcus neoformans*. *J. Infect. Dis.* 211 298–305. 10.1093/infdis/jiu441 25114160 PMC4342695

[B21] Donlan R. M. Costerton J. W. (2002). Biofilms: survival mechanisms of clinically relevant microorganisms. *Clin. Microbiol. Rev.* 15 167–193. 10.1128/CMR.15.2.167-193.2002 11932229 PMC118068

[B22] Feder V. Kmetzsch L. Staats C. C. Vidal-Figueiredo N. Ligabue-Braun R. Carlini C. R. (2015). *Cryptococcus gattii* urease as a virulence factor and the relevance of enzymatic activity in cryptococcosis pathogenesis. *FEBS J.* 282 1406–1418. 10.1111/febs.13229 25675897

[B23] Firacative C. Duan S. Meyer W. (2014). *Galleria mellonella* model identifies highly virulent strains among all major molecular types of *Cryptococcus gattii*. *PLoS ONE* 9:e105076 10.1371/journal.pone.0105076 PMC4136835 25133687

[B24] Fuchs B. B. Mylonakis E. (2006). Using non-mammalian hosts to study fungal virulence and host defense. *Curr. Opin. Microbiol.* 9 346–351. 10.1016/j.mib.2006.06.004 16814595

[B25] Fuchs B. B. O. Brien E. Khoury J. B. Mylonakis E. (2010). Methods for using *Galleria mellonella*as a model host to study fungal pathogenesis. *Virulence* 1 475–482. 10.4161/viru.1.6.12985 21178491

[B26] García-Rivera J. Chang Y. C. Kwon-Chung K. J. Casadevall A. (2004). *Cryptococcus neoformans* CAP59 (or Cap59p) is involved in the extracellular trafficking of capsular glucuronoxylomannan. *Eukaryot. Cell* 3 385–392. 10.1128/EC.3.2.385-392.2004 15075268 PMC387637

[B27] Grijpstra J. Gerwig G. J. Wösten H. Kamerling J. P. de Cock H. (2009). Production of extracellular polysaccharides by CAP mutants of *Cryptococcus neoformans*. *Eukaryot. Cell* 8 1165–1173. 10.1128/EC.00013-09 19542308 PMC2725565

[B28] Hall-Stoodley L. Costerton J. W. Stoodley P. (2004). Bacterial biofilms: from the natural environment to infectious diseases. *Nat. Rev. Microbiol.* 2 95–108. 10.1038/nrmicro821 15040259

[B29] Hicks J. K. Heitman J. (2007). Divergence of protein kinase A catalytic subunits in *Cryptococcus neoformans* and *Cryptococcus gattii* illustrates evolutionary reconfiguration of a signaling cascade. *Eukaryot. Cell* 6 413–420. 10.1128/EC.00213-06 17189488 PMC1828938

[B30] Kavanagh K. Reeves E. P. (2004). Exploiting the potential of insects for in vivo pathogenicity testing of microbial pathogens. *FEMS Microbiol. Rev.* 28 101–112. 10.1016/j.femsre.2003.09.002 14975532

[B31] Kraus P. R. Fox D. S. Cox G. M. Heitman J. (2003). The *Cryptococcus neoformans* MAP kinase Mpk1 regulates cell integrity in response to antifungal drugs and loss of calcineurin function. *Mol. Microbiol.* 48 1377–1387. 10.1046/j.1365-2958.2003.03508.x 12787363 PMC1635492

[B32] Kronstad J. W. Attarian R. Cadieux B. Choi J. D’Souza C. A. Griffiths E. J. (2011). Expanding fungal pathogenesis: *Cryptococcus* breaks out of the opportunistic box. *Nat. Rev. Microbiol.* 9 193–203. 10.1038/nrmicro2522 21326274 PMC4698337

[B33] Kwon-Chung K. J. Edman J. C. Wickes B. L. (1992). Genetic association of mating types and virulence in *Cryptococcus neoformans*. *Infect. Immun.* 60 602–605. 1730495 10.1128/iai.60.2.602-605.1992 PMC257671

[B34] Kwon-Chung K. J. Polacheck I. Bennett J. E. (1982). Improved diagnostic medium for separation of *Cryptococcus neoformans* var. neoformans (serotypes A and D) and *Cryptococcus neoformans* var. gattii(serotypes B and C). *J. Clin. Microbiol.* 15 535–537. 7042750 10.1128/jcm.15.3.535-537.1982 PMC272134

[B35] Livak K. J. Schmittgen T. D. (2001). Analysis of relative gene expression data using real-time quantitative PCR and the 2(-Delta DeltaC(T)) Method. *Methods* 25 402–408. 10.1006/meth.2001.1262 11846609

[B36] London R. Orozco B. S. Mylonakis E. (2006). The pursuit of cryptococcal pathogenesis: heterologous hosts and the study of cryptococcal host-pathogen interactions. *FEMS Yeast Res.* 6 567–573. 10.1111/j.1567-1364.2006.00056.x 16696652

[B37] Martinez L. R. Bryan R. A. Apostolidis C. Morgenstern A. Casadevall A. Dadachova E. (2006). Antibody-guided alpha radiation effectively damages fungal biofilms. *Antimicrob. Agents Chemother.* 50 2132–2136. 10.1128/AAC.00120-06 16723575 PMC1479110

[B38] Martinez L. R. Casadevall A. (2005). Specific antibody can prevent fungal biofilm formation and this effect correlates with protective efficacy. *Infect. Immun.* 73 6350–6362. 10.1128/IAI.73.10.6350-6362.2005 16177306 PMC1230912

[B39] Martinez L. R. Casadevall A. (2006a). *Cryptococcus neoformans* cells in biofilms are less susceptible than planktonic cells to antimicrobial molecules produced by the innate immune system. *Infect. Immun.* 74 6118–6123. 10.1128/IAI.00995-06 17057089 PMC1695499

[B40] Martinez L. R. Casadevall A. (2006b). Susceptibility of *Cryptococcus neoformans* biofilms to antifungal agents in vitro. *Antimicrob. Agents Chemother.* 50 1021–1033. 10.1128/AAC.50.3.1021-1033.2006 16495265 PMC1426450

[B41] Martinez L. R. Casadevall A. (2015). Biofilm formation by *Cryptococcus neoformans*. *Microbiol. Spectr.* 3 1–11. 10.1128/microbiolspec.MB-0006-2014 26185073

[B42] McClelland E. E. Hobbs L. M. Rivera J. Casadevall A. Potts W. K. Smith J. M. (2013). The role of host gender in the pathogenesis of *Cryptococcus neoformans* infections. *PLoS ONE* 8:e63632 10.1371/journal.pone.0063632 PMC3669355 23741297

[B43] Mendes-Giannini M. J. Taylor M. L. Bouchara J. B. Burger E. Calich V. L. Escalante E. D. (2000). Pathogenesis II: fungal responses to host responses: interaction of host cells with fungi. *Med. Mycol.* 38 113–123. 10.1080/mmy.38.1.113.123 11204137

[B44] Morrow C. A. Fraser J. A. (2013). Is the nickel-dependent urease complex of *Cryptococcus* the pathogen’s Achilles’ heel? *MBio* 4:e00408–e00413. 10.1128/mBio.00408-13 23800398 PMC3697809

[B45] Mylonakis E. Ausubel F. M. Perfect J. R. Heitman J. Calderwood S. B. (2002). Killing of *Caenorhabditis elegans* by *Cryptococcus neoformans* as a model of yeast pathogenesis. *Proc. Natl. Acad. Sci. U.S.A.* 99 15675–15680. 10.1073/pnas.232568599 12438649 PMC137775

[B46] Mylonakis E. Moreno R. El Khoury J. B. Idnurm A. Heitman J. Calderwood S. B. (2005). *Galleria mellonella* as a model system to study *Cryptococcus neoformans* pathogenesis. *Infect. Immun.* 73 3842–3850. 10.1128/IAI.73.7.3842-3850.2005 15972469 PMC1168598

[B47] Ngamskulrungroj P. Himmelreich U. Breger J. A. Wilson C. Chayakulkeeree M. Krockenberger M. B. (2009). The trehalose synthesis pathway is an integral part of the virulence composite for *Cryptococcus gattii*. *Infect. Immun.* 77 4584–4596. 10.1128/IAI.00565-09 19651856 PMC2747965

[B48] Perfect J. R. (2005). *Cryptococcus neoformans*: a sugar-coated killer with designer genes. *FEMS Immunol. Med. Microbiol.* 45 395–404. 10.1016/j.femsim.2005.06.005 16055314

[B49] Perfect J. R. (2013). Fungal diagnosis: how do we do it and can we do better? *Curr. Med. Res. Opin.* 29 3–11. 10.1185/03007995.2012.761134 23621588

[B50] Prates R. A. Fuchs B. B. Mizuno K. Naqvi Q. Kato I. T. Ribeiro M. S. (2013). Effect of virulence factors on the photodynamic inactivation of *Cryptococcus neoformans*. *PLoS ONE* 8:e54387 10.1371/journal.pone.0054387 PMC3548784 23349872

[B51] Pyrgos V. Seitz A. E. Steiner C. A. Prevots D. R. Williamson P. R. (2013). Epidemiology of cryptococcal meningitis in the US: 1997-2009. *PLoS ONE* 8:e56269 10.1371/journal.pone.0056269 PMC3574138 23457543

[B52] Qiu Y. Davis M. J. Dayrit J. K. Hadd Z. Meister D. L. Osterholzer J. J. (2012). Immune modulation mediated by cryptococcallaccase promotes pulmonary growth and brain dissemination of virulent *Cryptococcus neoformans* in mice. *PLoS ONE* 7:e47853 10.1371/journal.pone.0047853 PMC3478276 23110112

[B53] Rajasingham R. Boulware D. R. (2015). HIV care: ART adherence support and cryptococcal screening. *Lancet* 385 2128–2129. 10.1016/S0140-6736(15)60455-X 25765697

[B54] Ramage G. Rajendran R. Sherry L. Williams C. (2012). Fungal biofilm resistance. *Int. J. Microbiol.* 2012 528521 10.1155/2012/528521 PMC3299327 22518145

[B55] Ramage G. Williams C. (2013). The clinical importance of fungal biofilms. *Adv. Appl. Microbiol.* 84 27–83. 10.1016/B978-0-12-407673-0.00002-3 23763758

[B56] Robertson E. J. Casadevall A. (2009). Antibody-mediated immobilization of *Cryptococcus neoformans* promotes biofilm formation. *Appl. Environ. Microbiol.* 75 2528–2533. 10.1128/AEM.02846-08 19251903 PMC2675221

[B57] Sardi J. C. Pitangui N. S. Rodríguez-Arellanes G. Taylor M. L. Fusco-Almeida A. M. Mendes-Giannini M. J. (2014). Highlights in pathogenic fungal biofilms. *Rev. Iberoam. Micol.* 31 22–29. 10.1016/j.riam.2013.09.014 24252828

[B58] Sardi J. C. Scorzoni L. Bernardi T. Fusco-Almeida A. M. Mendes-Giannini M. J. (2013). *Candida* species: current epidemiology, pathogenicity, biofilm formation, natural antifungal products and new therapeutic options. *J. Med. Microbiol.* 62 10–24. 10.1099/jmm.0.045054-0 23180477

[B59] Scorzoni L. de Lucas M. P. Mesa-Arango A. C. Fusco-Almeida A. M. Lozano E. Cuenca-Estrella M. (2013). Antifungal efficacy during *Candida krusei* infection in non-conventional models correlates with the yeast in vitro susceptibility profile. *PLoS ONE* 8:e60047 10.1371/journal.pone.0060047 PMC3610750 23555877

[B60] Smith J. A. Kauffman C. A. (2012). Pulmonary fungal infections. *Respirology* 17 913–926. 10.1111/j.1440-1843.2012.02150.x 22335254

[B61] Sorrell T. C. (2001). *Cryptococcus neoformans* variety gattii. *Med. Mycol.* 39 155–168. 10.1080/mmy.39.2.155.168 11346263

[B62] Stie J. Fox D. (2012). Induction of brain microvascular endothelial cell urokinase expression by *Cryptococcus neoformans* facilitates blood-brain barrier invasion. *PLoS ONE* 7:e49402 10.1371/journal.pone.0049402 PMC3493525 23145170

[B63] Tortorano A. M. Dho G. Prigitano A. Breda G. Grancini A. Emmi V. (2012). ECMM-FIMUA Study Group. Invasive fungal infections in the intensive care unit: a multicentre, prospective, observational study in Italy (2006-2008). *Mycoses* 55 73–79. 10.1111/j.1439-0507.2011.02044.x 21668521

[B64] Trevijano-Contador N. Herrero-Fernández I. García-Barbazán I. Scorzoni L. Rueda C. Rossi S. A. (2015). *Cryptococcus neoformans* induces antimicrobial responses and behaves as a facultative intracellular pathogen in the non mammalian model *Galleria mellonella*. *Virulence* 6 66–74. 10.4161/21505594.2014.986412 25531532 PMC4603429

[B65] Uppuluri P. Lopez-Ribot J. L. (2010). An easy and economical in vitro method for the formation of *Candida albicans* biofilms under continuous conditions of flow. *Virulence* 1 483–487. 10.4161/viru.1.6.13186 21178492 PMC3073357

[B66] Walsh T. J. Schlegel R. Moody M. M. Costerton J. W. Salcman M. (1986). Ventriculoatrial shunt infection due to *Cryptococcus neoformans*: an ultrastructural and quantitative microbiological study. *Neurosurgery* 18 373–375. 10.1097/00006123-198603000-00025 3517675

[B67] Wand M. E. Bock L. J. Turton J. F. Nugent P. G. Sutton J. M. (2012). *Acinetobacter baumannii* virulence is enhanced in *Galleria mellonella* following biofilm adaptation. *J. Med. Microbiol.* 61 470–477. 10.1099/jmm.0.037523-0 22194338

[B68] Williams D. L. Costerton J. W. (2012). Using biofilms as initial inocula in animal models of biofilm-related infections. *J. Biomed. Mater. Res. B Appl. Biomater.* 100 1163–1169. 10.1002/jbm.b.31979 22120924

[B69] Williamson P. R. (1994). Biochemical and molecular characterization of the diphenol oxidase of *Cryptococcus neoformans*: identification as a laccase. *J. Bacteriol.* 176 656–664. 8300520 10.1128/jb.176.3.656-664.1994 PMC205102

[B70] Wright L. C. Payne J. Santangelo R. T. Simpanya M. F. Chen S. C. Widmer F. (2004). Cryptococcal phospholipases: a novel lysophospholipase discovered in the pathogenic fungus *Cryptococcus gattii*. *Biochem. J.* 384 377–384. 10.1042/BJ20041079 15320865 PMC1134121

[B71] Zaragoza O. Rodrigues M. L. de Jesus M. Frases S. Dadachova E. Casadevall A. (2009). The capsule of the fungal pathogen *Cryptococcus neoformans*. *Adv. Appl. Microbiol.* 6 133–216. 10.1016/S0065-2164(09)01204-0 19426855 PMC2739887

[B72] Zhu F. Ma X. H. Qin C. Tao L. Liu X. Shi Z. (2012). Drug discovery prospect from untapped species: indications from approved natural product drugs. *PLoS ONE* 7:e39782 10.1371/journal.pone.0039782 PMC3394748 22808057

[B73] Zhu X. Gibbons J. Garcia-Rivera J. Casadevall A. Williamson P. R. (2001). Laccase of *Cryptococcus neoformans* is a cell wall-associated virulence factor. *Infect. Immun.* 69 5589–5596. 10.1128/IAI.69.9.5589-5596.2001 11500433 PMC98673

