# Supplementary material for: Virulence of Cryptococcus sp. Biofilms In Vitro and In Vivo using Galleria mellonella as an Alternative Model
Source: Front Microbiol. 2016 Mar 9;7:290. doi: 10.3389/fmicb.2016.00290 (PMC4783715; doi:10.3389/fmicb.2016.00290)
Supplement: TABLE S1 — Primers used for RT-pPCR analysis for the virulence analysis. [file Table_1.DOC]

| **Genes** | | **Primers** |
| --- | --- | --- |
| ***C. neoformans*** |  |  |
|  | *CAP59* | Forward  5’-GATCTGGGAGCGCTGTAGAC-3’  Reverse  3’-TCTCTGCTCGTCGGTATCCT-5’ |
|  | *LAC1* | Forward  5’-GCCATCAGTCAGGCAGTT-3’  Reverse  3’-TCGGCCATGGAATTGGAAT-5’ |
|  | *URE1* | Forward  5’-TCGTATCGGTGAAGTCGTCACT-3’  Reverse  3’-GGACCACGGAATTGCTTCAT-5’ |
|  | *GADPH* | Forward  5’-TTTCCCGCGACTTTTTGG-3’  Reverse  3’-TCGCAGCCGAGTCTACGAT-5’ |
| ***C. gattii*** |  |  |
|  | *CAP59* | Forward  5’-CCTCCATATCCTCGACCTCA-3’  Reverse  3’-AGTTGGACCACGGTTTCTTG-5’ |
|  | *LAC1* | Forward  5’-TCCCTACGAGAGCACATTGGT-3’  Reverse  3’-CCCCTGCCCCCATGTC-5’ |
|  | *URE1* | Forward  5’-GCCGGACGTCATTGACTTTT-3’  Reverse  3’-CCCCAGTCGTTGGTCCAA-5’ |
|  | *GADPH* | Forward  5’-GGACCTCGTGCGCTTTATTC -3’  Reverse  3’-GCGGTAGGAGTCGGCACTT-5’ |
