# Supplementary material for: Virulence of Cryptococcus sp. Biofilms In Vitro and In Vivo using Galleria mellonella as an Alternative Model
Source: Front Microbiol. 2016 Mar 9;7:290. doi: 10.3389/fmicb.2016.00290 (PMC4783715; doi:10.3389/fmicb.2016.00290)
Supplement: TABLE S2 — Fungicidal concentration of amphotericin B for planktonic cells and biofilm madure of Cryptococcus neoformans and Cryptococcus gattii. [file Table_2.DOC]

| **Strains** | **Planktonic cells** | **Biofilm** |
| --- | --- | --- |
| *C. neoformans* 90012 | 0.5 µg/mL | 64.0 µg/mL |
| *C. gattii* 56990 | 0.25 µg/mL | 64.0 µg/mL |
